# Creation and Detection of Optical Spin in a Coupled Emitter–Plasmon System

**DOI:** 10.1021/acs.nanolett.5c05644

**Published:** 2026-02-09

**Authors:** Yining Xuan, Daito Miyazaki, Yuki Ishikawa, Hiromi Okamoto, Mark Sadgrove

**Affiliations:** † Department of Physics, 26413 Tokyo University of Science, 1-3 Kagurazaka, Shinjuku-ku, Tokyo 162-8601, Japan; ‡ Institute for Molecular Science, National Institutes of Natural Sciences, 38 Nishigonaka, Myodaiji, Okazaki, 444-8585 Aichi, Japan

## Abstract

We investigate the system of a linearly polarized dipole emitter coupled to the plasmonic modes of a gold nanorod (GNR). We show numerically that asymmetrical placement of the emitter relative to the GNR axes gives rise to a net optical spin for the field inside the rod, despite the apparent achirality of the system. We experimentally demonstrate this effect using electron beam excitation to create an effective point dipole emitter and coupling luminescence evanescently to a nanofiber probe which supports spin-momentum locked light. This converts the net spin of the field into a net directionality of propagation in the fiber modes, allowing detection in the far field.

## Graphical abstract

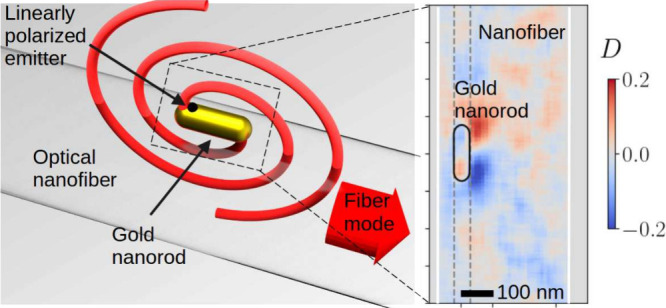

The production of photons with an enhanced generation rate and a desired spectrum is ably handled by cavity QED techniques, in particular the Purcell effect.^1,2^ By coupling photons from an emitter to a resonant mode, for which both the coupling *and* mode decay occur much faster than the rate at which photons are emitted into free space, the overall emission of the combined system takes on the characteristics of the mode itself, which may be engineered by nanofabrication. In recent years, it has been shown that photon polarization can also be engineered if the characteristics of the resonator mode can be suitably tailored. In particular, appropriate breaking of resonator symmetries, can produce polarized emission by either large resonator birefringence,^3–11^ asymmetrical pumping,^12,13^ or the use of chiral nanostructures.^14,15^ Because control of polarization allows control of propagation direction in chiral quantum optics, polarization control can also be applied to the routing of photons.^15–19^ In systems supporting plasmonic resonances, it has been shown that even for an achiral particle, such as a gold nanorod (GNR), optical chirality, including its simplest incarnation as circular polarization, can exist in the near field.^20–22^ Also relevant is the finding that even a linear dipole has a circularly polarized component in the near field which can be converted to a far-field circular polarization by appropriate coupling techniques.^23^ We note that, in the cases above, the experimental demonstration was achieved via external laser excitation of the plasmonic modes of a metal nanoparticle with a specific polarization.

The convenience that would be afforded by being able to use a simple, nonchiral structure to induce circularly polarized photon emission from a linearly polarized point emitter is a strong motivation for further studies in this area. In this work, we investigate the system of a point emitter coupled to the surface plasmon modes (SPMs) of a GNR, with asymmetric emitter placement relative to the principle axes of the GNR, as depicted in Figure 1(a). We show that for dipole excitation centered either inside or outside the GNR, this leads to an asymmetrical distribution of optical spin within the GNR, despite the fact that the GNR’s localized surface plasmon modes are linearly polarized. Experimentally, we use a novel technique to confirm this effect by coupling electron beam-induced luminescence from the GNR evanescently using an optical nanofiber probe upon which the GNR is placed. The well-known spin-momentum locking property of the nanofiber modes^16,17,25,26^ transforms net optical spin into directionality of coupling to the fiber modes, allowing the spin to be inferred from intensity measurements at the fiber outputs.

**Figure 1:**
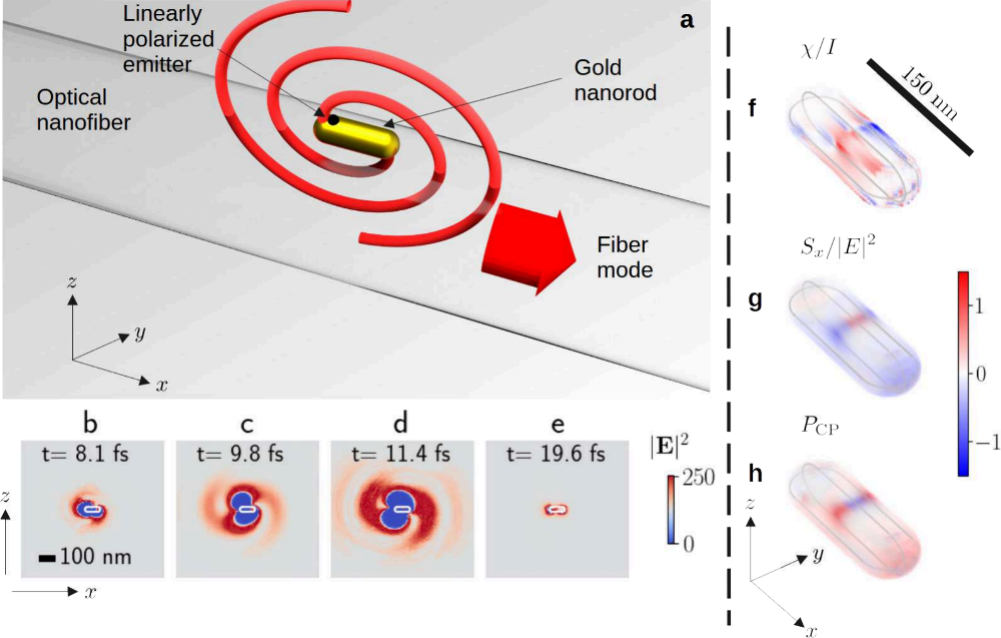
(a) Schematic illustration of the experiment. The black hemisphere represents the emitter, which is assumed to be *z*-polarized. The emitter couples to the plasmon modes of the GNR giving rise to a net rotating dipole moment. This leads to directional coupling to the optical nanofiber modes, as indicated by the red arrow. (b–e) Simulated near-field intensity distributions for a *z*-polarized dipole source to a GNR, at times as indicated in the top of each frame. The first frame shows a scale bar which is the same for all images. The GNR outline is shown in white. (f–h) normalized field chirality χ/*I*, normalized *y*-component of the field spin *S*_*y*_/|*E*|^2^, and degree of polarization *P*_CP_, respectively. All quantities are shown within the rod volume. The outline of the rod in the *x*–*z* and *x*–*y* planes is shown in gray in each case. The GNR material is a preset which uses an index profile based on optical measurements.^24^

We first examine the phenomenon under consideration by using snapshots of the near-field distribution generated by finite-difference time-domain (FDTD) simulations. Throughout, we consider a GNR of length 150 nm and diameter 50 nm, and an optical wavelength of 600 nm. Details of simulation convergence are given in Supplementary Figure S1. The emitter is modeled by a point dipole current source with linear *z*-polarization, and a Gaussian time envelope, with a total duration of ∼5 fs. Results shown in Figures 1(b-d) are for a dipole placement (*x*_*e*_, *y*_*e*_, *z*_*e*_) = (−50, 0, 15) nm, where the origin is taken to be the GNR center, and the subscript *e* denotes that the coordinates are those of the emitter. Initially, the results demonstrate the characteristic two-armed spiral in the near-field associated with a circularly polarized electric dipole. (See Supplementary Figure S2 for a comparison with a circularly polarized point emitter). Nearer to the end of the plasmon lifetime, a typical dipolar distribution about the GNR caps is observed (Figure 1(e)). Details of the plasmon mode properties are found in Supporting Information Table S1, along with simulations exploring various parameter variations in Supporting Information Figures S3, S4, and S5.

The observed time domain behavior of the electric field evinces the existence of optical chirality in its simplest form - i.e. circular polarization or spin. The modern understanding of optical chirality is based on the chirality density χ along with the optical spin density **S**. For the chirality density, it is sufficient to use the definition

1 $$\chi =Im\left\{E^{*}\cdot H\right\}$$

for electric (magnetic) field **E** (**H**) giving a quantity with units of power per unit area.

The optical spin associated with the electric field is proportional to

2 $$S=Im\left\{E^{*}\times E\right\}$$

a quantity with units V^2^m^–2^. From here on, we will consider the dimensionless chirality density χ/*I*, where *I* = 2*ε*_0_ *c*|*E*|^2^, and the dimensionless spin density **S**/|**E**|^2^. These normalized quantities are useful, because the unnormalized densities are dominated by numerical fluctuations in the polarization in the high intensity region near the emitter. The normalized quantities allow us to investigate the polarization of the plasmon field itself, which is our actual area of interest.

Lastly, we also consider a more intuitive quantity, the degree of circular polarization (DCP) which is given by^21^

3 $$P_{CP}=\frac{\left|E^{*}\cdot u_{L}{|}^{2}-\right|E^{*}\cdot u_{R}{|}^{2}}{\left|E^{*}\cdot u_{L}{|}^{2}+\right|E^{*}\cdot u_{R}{|}^{2}}$$

where $u_{L}=\left(e_{x}+ie_{z}\right)/\sqrt{2}$ is the polarization vector for left-hand circular polarized (LCP) light which rotates counterclockwise in time using the phase convention exp­[*i*(**k**·**r** – *ωt*)]. The right-hand circular polarization (RCP) vector is $u_{R}=u_{L}^{*}$ . Taking the direction of propagation as **e**_*z*_ × **e**_*x*_ = **e**_*y*_ makes this definition coincide with that of the standard Jones calculus.^27^

We will use the time-averaged fields from simulations to evaluate the above quantities. In Figure 1(f), the normalized chirality density is shown over the volume of the GNR for an emitter placed at position (*x*_*e*_, *y*_*e*_, *z*_*e*_) = (−50, 0, 15) nm. Although the chirality density is generally nonzero, it is crucial to note that its structure has sign flipped mirror symmetry in the *x*-*z* plane. That is, every positive region is exactly matched by a negative region of the same size and shape under reflection. For this reason, the average value over the GNR volume vanishes. On the other hand, the distribution of *S*_*y*_/|**E**|^2^ shown in Figure 1(g) does not have this property, showing a net negative value after volume averaging. (The distributions for *S*_*x*_ and *S*_*z*_ *do* exhibit the same sign-flipped mirror symmetry as the chirality density and thus also average to zero, hence their omission here).

Lastly, we plot *P*_CP_ in Figure 1(h). We see that it has a similar distribution to the *y* – component of the spin density, with its net positive value indicating a dominant left-hand circular polarization. Indeed, we note that if *E*_*y*_ is small compared to *E*_*x*_ and *E*_*z*_, it can be shown that *P*_CP_ ≈ – *S*_*y*_/|**E**|^2^. For this reason, in the following theoretical exposition, we choose to present results for *P*_CP_ alone, since it is arguably the more intuitive measure of circular polarization, and is simpler to relate to the experimentally measured directionality.

In addition, we note that all the quantities defined so far depend on the emitter position **r**_*e*_, and the position **r** where the quantity is evaluated within the rod. In the current study we are able to vary **r**_*e*_ systematically, but our intensity signal is effectively integrated over **r**. Thus, it is useful to define the integrated quantity

4 $$\left\langle P_{CP}\left(r_{e}\right)\right\rangle =\frac{\int_{V}d^{3}r\left(|E^{*}\cdot u_{L}{|}^{2}-|E^{*}\cdot u_{R}{|}^{2}\right)}{\int_{V}d^{3}r\left(|E^{*}\cdot u_{L}{|}^{2}+|E^{*}\cdot u_{R}{|}^{2}\right)}$$

We will typically suppress the **r**_*e*_ dependence in the notation used below.

In Figure 2, we investigate the behavior revealed above in more detail. First, in Figures 2(a-c), we investigate the value of *P*_CP_ for an emitter position that is unshifted relative to at least one of the principle axes of the GNR. In each case, the result is a *P*_CP_ distribution which shows sign flipped mirror symmetry with respect to at least one plane and thus there is zero degree of circular polarization when averaged over the entire GNR volume, as indicated by the vanishing values of ⟨*P*_CP_⟩ shown for each figure.

**Figure 2:**
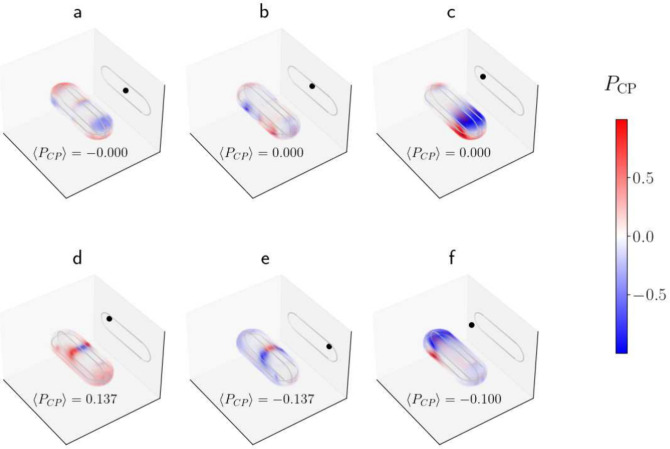
(a–g) Simulated *P*_CP_ distributions over the GNR volume for emitter positions (*x*_*e*_, *y*_*e*_, *z*_*e*_) = (0, 0, 0), (0, 0, 15), (−50, 0, 0), (−50, 0, 15), (50, 0, 15), and (−85, 0, −35), respectively. (All positions are given in nm.) In all simulations, the GNR length was 150 nm and the diameter was 50 nm. The dipole emitter was set to be *z*-polarized, with a center wavelength of 600 nm. The GNR material is a preset which uses an index profile based on optical measurements.^24^

However, if the dipole is displaced from the center along *both* the *x* and *z* axes, then a certain polarization will dominate, as seen in Figures 2(d) and (e). Figure 2(f) demonstrates that the effect also holds for a dipole coupled from outside the GNR, so long as displacement along both principle axes is present. In particular, Figures 2(d) and (e) demonstrate that the direction of displacement along the *x*-axis (for a set *z* value) changes the sign of ⟨*P*_CP_⟩, i.e., the handedness of the net polarization. The same is true for displacement along the *z* axis as may be inferred from Figure 2(f). In our experimental implementation, the *z* position of the emitter is determined by geometry as will be explained later, leading to our particular choice of position for the emitter placed outside the GNR in Figure 2(f).

In the present work, due to the lack of an analytical method to reproduce the numerical results above, we will avoid applying a particular qualitative interpretation to the results, aiming instead to reproduce the numerical results experimentally. Let us briefly remark that the dominant polarization seen in Figures 2(d–f) can be reproduced by considering the GNR to be a short nano*wire*, and considering the local polarization of the nanowire modes. A more detailed analysis is beyond the scope of the present Letter.

Taken together, the results shown in Figures 1 and 2 demonstrate numerically the existence of a circularly polarized field component inside the GNR, despite the fact that the emitter is linearly polarized. In turn, the circularly polarized field induces a dipole moment with a circularly polarized component in the GNR, and this dipole moment can couple to the fiber modes, as well as produce emission with a circularly polarized component propagating along the *y* axis.

Let us move on to the principle of our experimental measurements. We use the cathode luminescence (CL) method to excite emission from a GNR using the electron beam of a scanning electron microscope (SEM). The electrons are assumed to induce a point dipole-like excitation when entering or passing very near to the GNR, due to the oscillating dipole moment they induce in the material. The validity of this assumption along with the consequence that the measured intensity is proportional to the photonic local density of states (LDOS) has been discussed extensively elsewhere.^28–30^ We have confirmed that for the case of an optical nanofiber, measured results agree well with LDOS predictions calculated for the fiber fundamental mode, and reproduce the radial dependence of the LDOS when the electron stopping distance is varied.^31^ These results suggest strongly that the current experiment can be simulated using a point dipole current source (polarized along the electron beam axis) placed at the expected stopping position of the electron.

As shown in Figure 3(a), an important novel aspect of the current experiment, relative to our previous nanofiber-based CL experiment,^31^ is that emitted luminescence is coupled to the evanescent field of the optical nanofiber (ONF), which is assumed to be aligned with the GNR axis.

**Figure 3:**
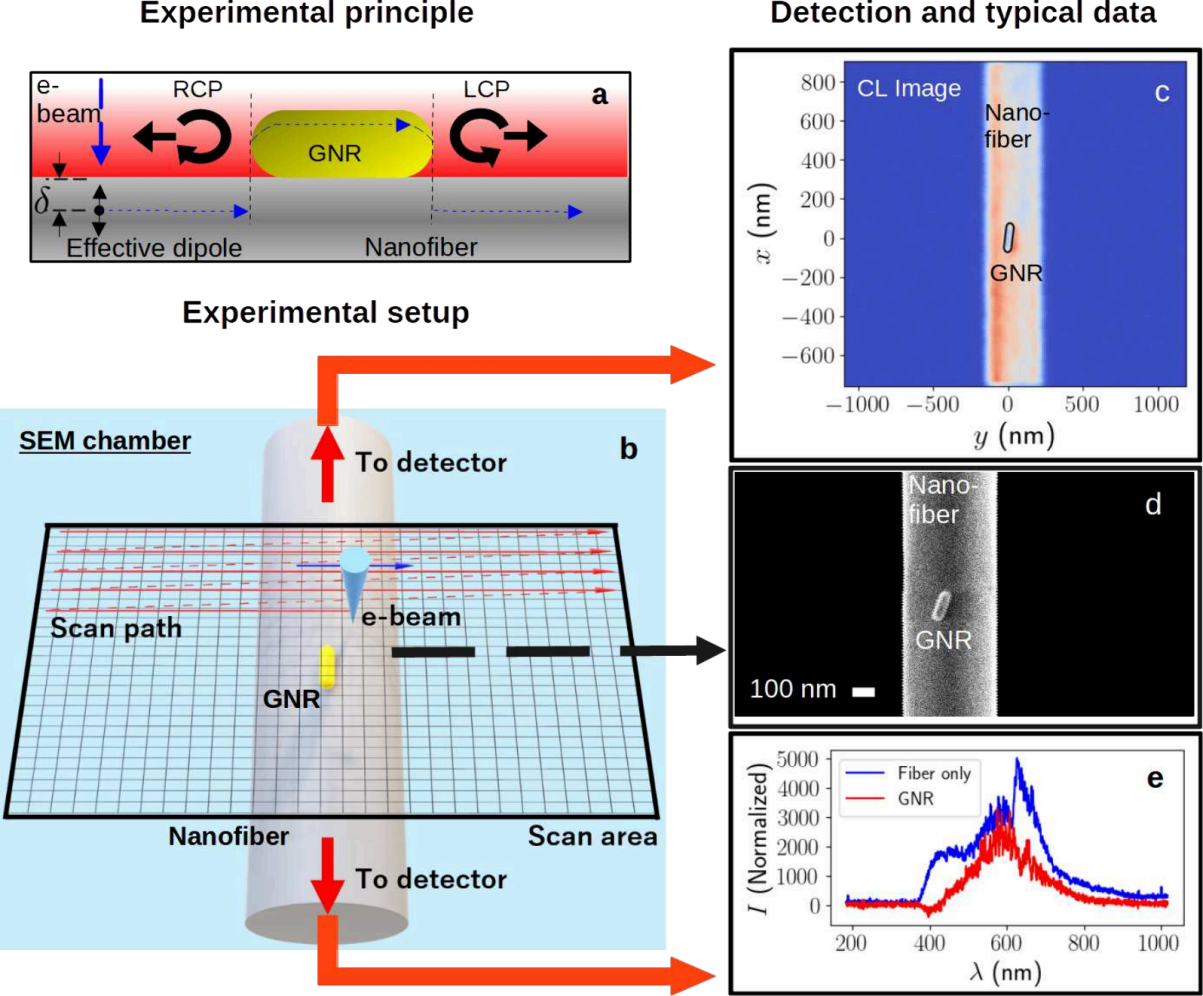
(a) Principle of the experiment. An electron beam is incident on the GNR, creating an effective dipole excitation at the position where the electron stops. CL from the so-excited GNR plasmon mode couples to the optical nanofiber fundamental mode with a directionality dependent on the circular polarization component of the induced dipole moment. (b) Experimental setup inside the SEM. A GNR deposited on an optical nanofiber is excited by an electron beam (diameter ≈ 5 nm). The resulting optical emission is coupled evanescently to the fiber and sent via a fiber feedthrough to a detector. (c) CL image from light detected at one end of the fiber using a single photon counting module (SPCM). (d) SEM image of the GNR and nanofiber from secondary electron detection. (e) Spectrum of the bare fiber (blue line) and the GNR (red line), as indicated in the legend, measured by an optical multichannel analyzer.

Our numerical calculations automatically include the effect of the induced polarization in the GNR and the resultant coupling to the nanofiber. However, to develop an intuition for the type of behavior we might expect, let us make the following simple model of the induced dipole moment at a point **r** inside the GNR, and for an emitter position **r**_*e*_

5 $$P\left(r,r_{e}\right)=\alpha E\left(r,r_{e}\right)$$

The assumption of a scalar polarizability α is not as unrealistic as it might first appear, due to the fact that the wavelength we consider (600 nm) lies between the orthogonally polarized plasmon resonances of the GNR.

The intensity of coupling to a given mode ϵ is given by^32^ *I* ∝ |**P**(**r**_*e*_, **r**)*·**ϵ­(r)**|^2^. In the experiment, the intensity is measured for each emitter position **r**_*e*_, but the excited luminescence comes from the plasmon excitation of the entire GNR. Thus, the measured value corresponds to an intensity integrated over the position **r** within the rod. Using ϵ­(**r**)_±_ to represent the ± *x* propagating fiber modes, we thus define the directionality of coupling to the fiber modes

6 $$D\left(r_{e}\right)=\frac{\int_{V}d^{3}r\left(|P^{*}\cdot \epsilon_{+}{|}^{2}-|P^{*}\cdot \epsilon_{-}{|}^{2}\right)}{\int_{V}d^{3}r\left(|P^{*}\cdot \epsilon_{+}{|}^{2}+|P^{*}\cdot \epsilon_{-}{|}^{2}\right)}=\frac{\int_{V}d^{3}r\left(|E^{*}\cdot \epsilon_{+}{|}^{2}-|E^{*}\cdot \epsilon_{-}{|}^{2}\right)}{\int_{V}d^{3}r\left(|E^{*}\cdot \epsilon_{+}{|}^{2}+|E^{*}\cdot \epsilon_{-}{|}^{2}\right)}$$

where we have used eq 5 to reach the second expression on the right-hand side. We note that, within the GNR

$$D\approx \left\langle P_{CP}\right\rangle \approx -\frac{S_{y}}{|E{|}^{2}}$$

is true if the fiber modes are approximately circularly polarized in the *x* – *z* plane. That is, the spin-momentum locking in the fiber modes converts ⟨*P*_CP_⟩ at a certain emitter position to a directionality *D* which is ideally numerically equal. We note again that our numerical simulations do not use the approximations utilized to arrive at the above result, and the simple model of the local dipole moment is only used to establish expectations regarding the qualitative behavior.

Having established the principle of our technique for measuring *P*_CP_ we move on to the experimental setup. Figure 3(b) shows a schematic representation of our experiment. An optical nanofiber with a diameter of ∼500 nm is mounted in the sample chamber of a SEM (Carl Zeiss SUPRA50). The SEM has a beam diameter of approximately 5 nm^31^ which sets the lower limit on the resolution of both secondary electron images and CL scans. In all experiments, the acceleration voltage of the electrons was set to be 2 kV. The scan resolution was 2.8 nm in the *x* direction and 10.5 nm in the *y* direction.

A single GNR was deposited on the nanofiber surface using a micropipette.^33^ A fiber feedthrough allowed the collected CL to be measured using single photon counting modules (SPCMs) located outside the SEM,^31^ as shown in Figure 3(c). Aside from CL measurement by SPCM, it is also possible to take simultaneous measurements of secondary electrons to produce a standard SEM image (Figure 3(d)) and measurements of the CL spectrum using an optical multichannel analyzer (Kymera 193i, Newton DU970P-BVF). Figure 3(e) shows recorded spectra for the fiber alone (black curve) and for the GNR with the fiber background subtracted (red curve). We see that the peak CL wavelength occurs near 600 nm, and thus this wavelength was used in the simulation results shown so far.

We now turn to the main result of the paper - the demonstration of a circular polarization component or optical spin in the GNR’s induced dipole moment (and, by extension, in the emitted light) using spin-momentum locked CL measurements. Figure 4(a) shows the directionality calculated from the CL measurements associated with the sample seen in Figures 3(c-d) in a region surrounding the GNR. The associated simulation results are shown in Figure 4(b). We emphasize that within the GNR, the directionality can be interpreted as the degree of circular polarization or as minus the normalized optical spin along the *y* axis.

**Figure 4:**
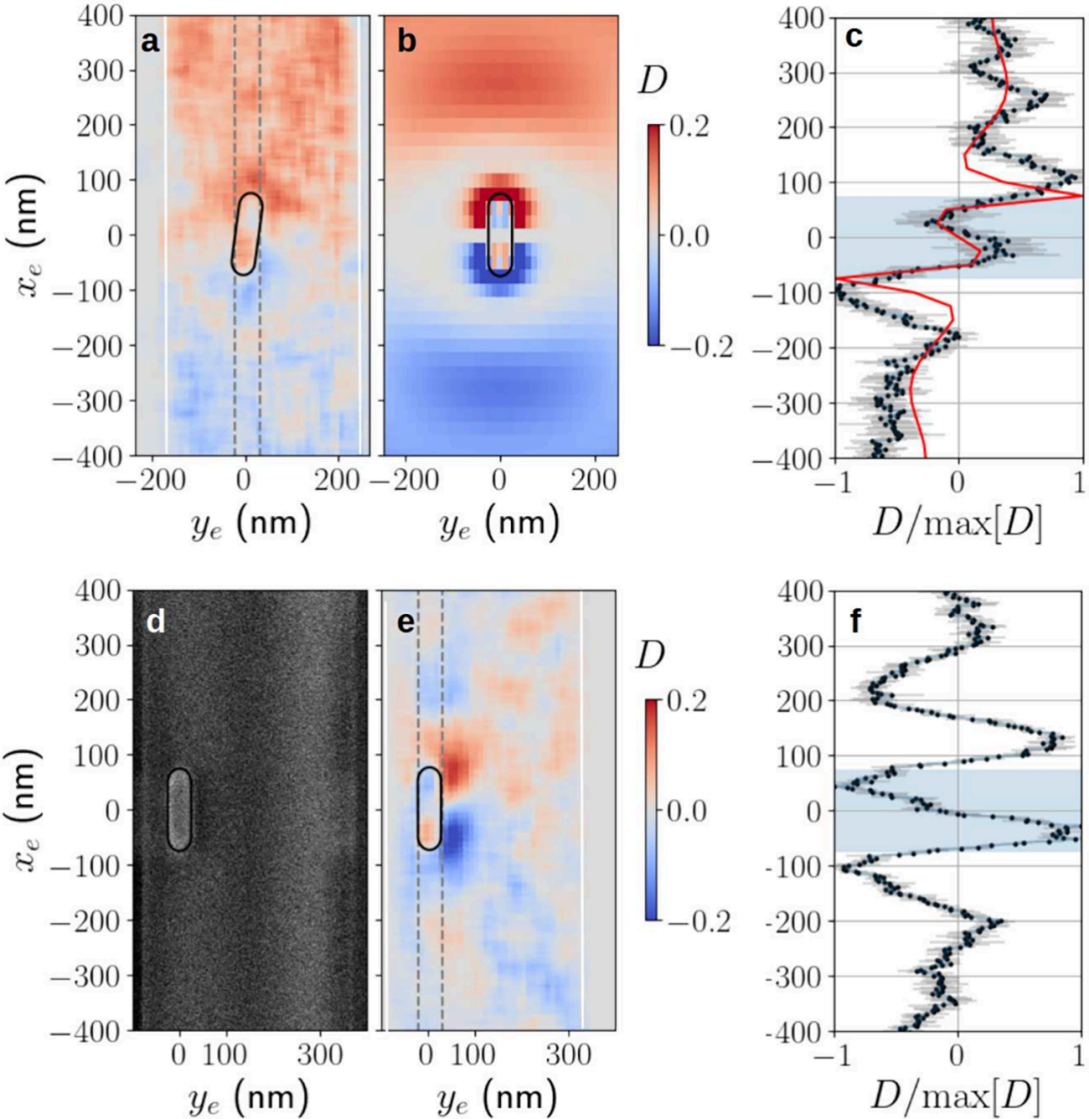
Experimental measurements of *D* for the GNR shown in Figure 3(d). (a) Experimentally measured directionality. Vertical white lines indicate the fiber edges. (b) Numerically calculated directionality. (c) Comparison of experimentally measured directionality integrated over the GNR width (black dots with gray error bars) with the numerically predicted value (red curve). (d–f) Similar measurements showing the effect of displacement of the GNR from the ONF center. (d) SEM image of the GNR sample used. (e) The measured directionality for the sample shown in panel d. (f) The averaged directionality over the region indicated by the gray dashed lines in panel e. In both cases the pale blue regions in panels c and f indicate the GNR region.

The experimental result demonstrates a number of features predicted by the detailed numerical simulations. First, there is the directionality inside the GNR which, as predicted, flips from positive to negative as *x*_*e*_ is increased past the origin, owing to a change in the dominant circular polarization from LCP to RCP.

Second, the structure of *D* outside the GNR is also reproduced. Most intriguingly, the reversal of directionality when the GNR boundary is crossed along with oscillations in *D* are apparent. These details are more clearly seen by looking at the behavior of *D* as a function of *x*_*e*_ with *y*_*e*_ = 0, as shown in Figure 4(c). To make this graph, the experimental data in Figure 4(a) was averaged over the region indicated by the gray dashed lines, and normalized to its maximum giving the black points shown, while the light gray error bars show the standard deviation over the same region. The red curve in Figure 4(c) shows the normalized simulation results corresponding to *y*_*e*_ = 0. We see good correspondence between the predicted and measured structure in *D*, including the reversal of sign, and position of peaks.

The directionality seen outside the GNR in both simulations and experiments must be interpreted with some care compared to that seen inside the GNR. This is because the source of the dipole excitation outside the GNR is the excitation of nonbridging oxygen hole centers (NBOHCs) in the fiber^31^ which couple to the GNR plasmon, but also experience interference due to scattering from the GNR. This leads to the oscillations in *D* seen outside the GNR. We also note that the center wavelength of NBOHCs in silica is about 650 nm, in comparison to the 600 nm center wavelength seen for the GNR CL spectrum. This wavelength difference is taken into account in our simulations.

A natural question is how the relative position of the GNR and fiber affects the results. In Figures 4(e),(f), we show measurements of *D* of a GNR which is near to the edge of the ONF, as seen in the SEM image in Figure 4(d). Although the distribution of *D* around the GNR is no longer symmetrical about the *x*_*e*_ axis, the same qualitative behavior seen in Figures 4(a-c) is reproduced. We note that our experimentally measured values of *D* approach the numerically calculated limit of 0.15.

In conclusion, we have numerically investigated and experimentally demonstrated the phenomenon of circularly polarized fields produced when a dipole source is coupled to the plasmonic modes of a GNR at a position displaced from its center. We tested this phenomenon experimentally by using an electron beam to produce an effective point dipole excitation in a GNR and collected the induced cathode luminescence evanescently, allowing the degree of circular polarization, or, equivalently the *y* component of the optical spin, to be converted to directionality of mode propagation, due to the spin-momentum locking of the nanofiber modes.

Although our results might seem at odds with established facts regarding the linear polarization of emitters coupled to anisotropic resonators,^3–8^ to the best of our knowledge, no previous studies have had the necessary control over emitter-particle positioning or the right particle geometry to discover the effect reported here. Some characteristics of the present study are arguably present in the work of Joos et al.^34^ where a GNR on a nanofiber was excited by a laser producing arbitrary polarizations of light in the fiber mode itself. However, no concept of emitter-GNR coupling or directionality was present there. In addition, although electron beam methods have been used to induce directional polarization from isotopic particles,^35^ this was not mode-resolved or correlated with circular polarization, as in the present study.

We have not discussed in detail the emitted electro-magnetic radiation associated with the induced circular dipole moment, as it was not detected directly in the current work. However, the simulations clearly predict emitted radiation with a significant circular polarization propagating along the positive and negative *y*-axes (see Supporting Information Figures S6 and S7). In integrated optics settings, use of coupling to a nonchiral nanostructure from a linearly polarized emitter could provide a simplified method to produce circularly polarized photons. Furthermore, we note that the realization of directionality of emission is also useful in itself, and has been the focus of many studies in the field of chiral quantum optics in recent years.^16^

Finally, we note that evanescent collection of CL demonstrated here is promising, as it sheds light on new aspects of cathode luminescence through the spin-momentum locking property. In this case, it allowed us to use directionality as a proxy for optical spin, which would have been difficult to measure with standard CL setups.

## Supplementary Material



## Data Availability

The raw data relevant to this paper and the computer code required to analyze it may be found in the following FigShare repository: 10.6084/m9.figshare.30382792 https://figshare.com/s/869e29b446a062aa27f0

## Notes

Supporting simulation results, supporting experimental results, simulation methods, experimental methods, data analysis methods (PDF)
