# Supplementary material for: Creation and Detection of Optical Spin in a Coupled Emitter–Plasmon System
Source: Nano Lett. 2026 Feb 9;26(6):2129–35. doi: 10.1021/acs.nanolett.5c05644 (PMC12922173; doi:10.1021/acs.nanolett.5c05644)
Supplement: Supplementary file 1 [file nl5c05644_si_001.pdf]

Supporting information for "Creation and detection of optical spin in a coupled emitter-plasmon system"

Yining Xuan,† Daito Miyazaki,† Yuki Ishikawa,† Hiromi Okamoto,‡ and Mark Sadgrove*,†

†*Department of Physics, Tokyo University of Science, 1-3 Kagurazaka Shinjuku-ku Tokyo 162-8601*

‡*Institute for Molecular Science, National Institutes of Natural Sciences, 38 Nishigonaka, Myodaiji, Okazaki, 444-8585, Aichi, Japan*

E-mail: mark.sadgrove@rs.tus.ac.jp

Abstract

We present supporting information for the paper "Creation and detection of optical spin in a coupled emitter-plasmon system".

Simulation methods

In this section we give details regarding our finite difference time domain (FDTD) simulations. All simulations were performed using a commercial FDTD solver.[1]

Simulation setup

In all our simulations, the gold nanorod is a hemisphere capped cylinder of total length 150 nm and radius 25 nm. The refractive index is set to the provided preset which uses complex index values taken from the CRC handbook of Chemistry and Physics.[2]

The gold nanorod is surrounded by a custom mesh area of volume 1 μm^3 in which a mesh size of 2 nm is set. Outside this area, the mesh size is left to the software to determine by setting a preset mesh level of 8. The source used for the simulations is a numerical approximation of a z-polarized point dipole current source, with a wavelength centered on 600 nm for excitation within the GNR. The placement is decided by the penetration depth δ of 2 keV electrons into a gold surface which we take as 10 nm.[3]

For simulations which include a nanofiber, the fiber material is set to a silica preset, with a refractive index of approximately 1.45 near 600 nm. The dipole position is decided by the penetration depth of 2 keV electrons into a silica surface which we take as 20 nm.[4]

Simulations were typically run until the remaining electromagnetic energy dropped to 0.001% of the initial energy.

Supporting simulation results - checks of simulation validity

Convergence of results with mesh density

In FDTD simulations, verifying the convergence with respect to mesh density is an essential step to ensure the reliability of numerical results. We systematically refined the mesh density to examine its influence on the calculated transmission and directionality. In each case, the dipole source was placed in the same position in the upper-right corner of the GNR. This configuration excites an RCP component in the dipole moment, which preferentially couples into the $-x$ direction, as recorded by power monitors placed along the $\pm x$ directions.

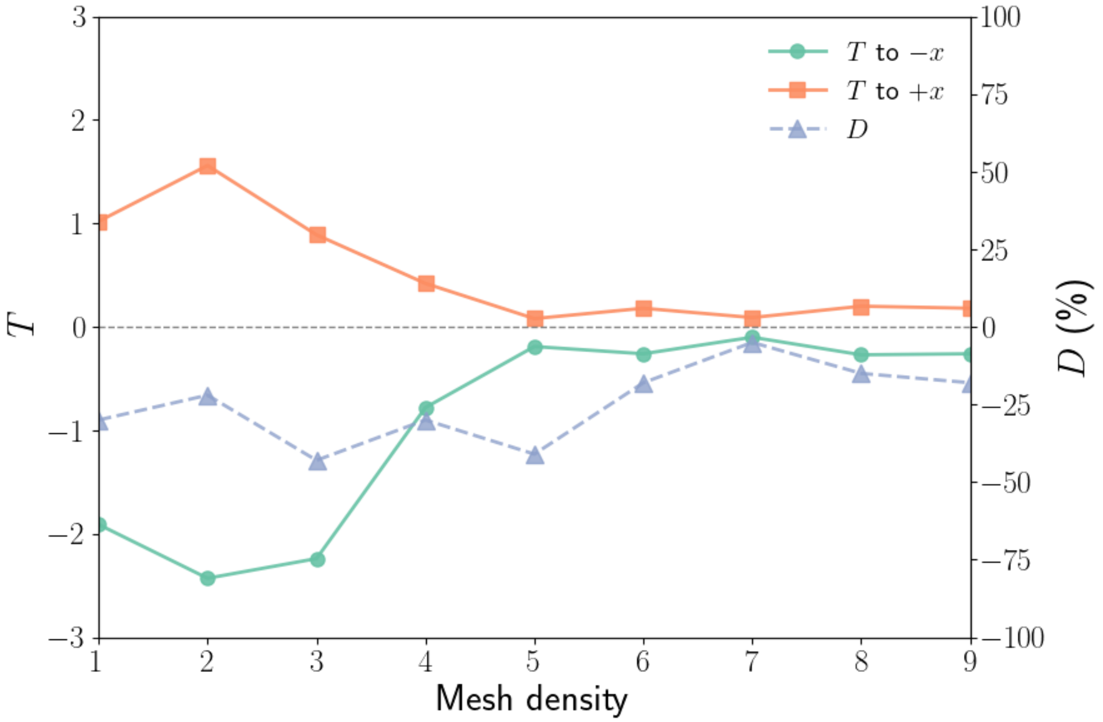

Figure S1: Transmission and directionality for a 145 nm × 50 nm GNR plasmon mode excited by a point dipole source located at the upper-right corner under different mesh densities. Levels 1–8 correspond to the default automatic mesh settings in FDTD (from coarsest to finest). Level 9 corresponds to mesh size 8 with an additional refined custom mesh of 2 nm applied around the GNR.

The results are shown in Fig. S1. Here, the horizontal axis (mesh density levels 1–8) corresponds to the default automatic mesh sizes defined in the FDTD software, where level 1 is the coarsest and level 8 the finest. Mesh density level 9 denotes a customized setting, in which the overall mesh was kept at level 8 while an additional refined mesh of 2 nm was applied locally in a 1 μm^2 volume centered on the GNR, as used in the simulations presented in the main paper. Although fluctuations in the parameters are not completely eliminated, the transmissions are seen to reduce steadily for mesh sizes between 2 and 5 after which they are distributed over a small range of values. Notably, the sign of the directionality is always negative as expected, but declines with refinement, saturating to a value close to 20%. This demonstrates satisfactory convergence and confirms the reliability of our simulation results.

Direct observation of rotating polarization in simulations

Circular polarization may be observed directly in the simulations by recording the fields at each time step, creating a movie. Representative snapshots under different conditions are presented in Fig. S2, and the corresponding video files are also available as Supporting Material.

As a reference, we first constructed ideal cases of left- and right-handed circular polarization (LCP and RCP) by simulating two orthogonal point dipole sources with a $\pm 90^\circ$ phase difference. The corresponding results are shown in Figs. S2a (LCP) and c (RCP). When the point dipole source is placed at the *upper-left* corner of the GNR, light with an LCP component is generated, as seen in Fig. S2b. Similarly, when the dipole source is located at the *upper-right* corner, an RCP component appears, as shown in Fig. S2d.

Figs. S2(e) and (f) present the case where the GNR was placed on the surface of an ONF, with the point dipole source positioned at the *upper-right* corner. According to the mechanism discussed in the main text, light with an RCP component is generated, as shown in Fig. S2(e). Subsequently, due to spin–momentum locking, the excitation couples directionally to the ONF as seen in Fig. S2(f).

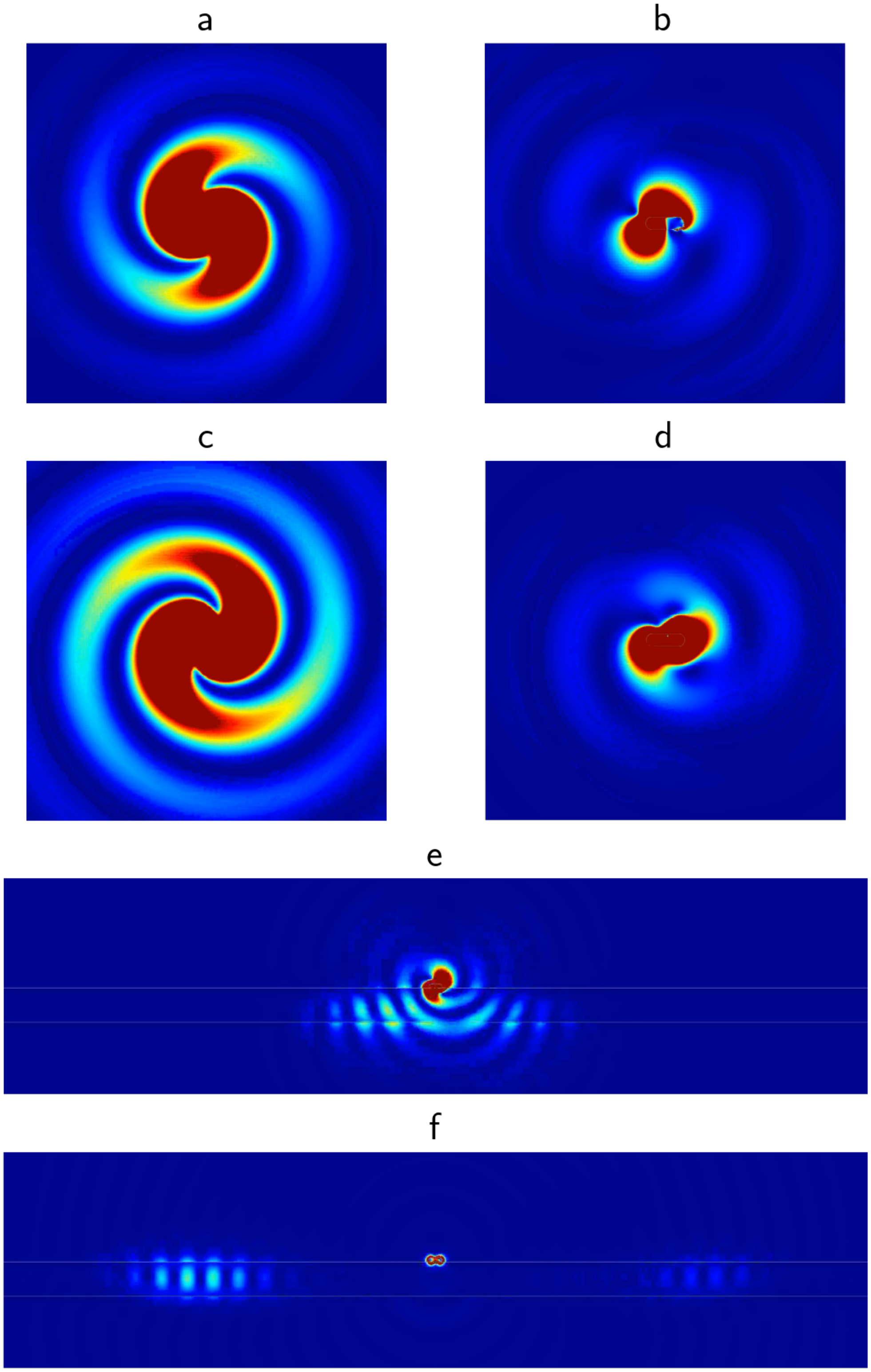

Figure S2: Snapshots from the FDTD movie monitor. (a) and (c) correspond to the ideal LCP and RCP cases constructed using two orthogonal dipole sources with a $\pm 90^{\circ}$ phase difference. (b) and (d) show results for a GNR with dimensions $150\,\mathrm{nm} \times 50\,\mathrm{nm}$. (e) and (f) show the case where the GNR is positioned on the surface of an ONF, and for which the dipole source was placed at the upper-right corner. (e) shows the initial generation of light with an RCP component, while (f) demonstrates the subsequent directional coupling into the ONF via spin–momentum locking.

Supporting simulation results - further investigation of the rotating dipole moment effect

In the following subsections, we provide further numerical evidence of the main finding of our paper - the existence of an induced rotating polarization which gives rise to chiral emission and directional coupling to the optical nanofiber.

GNR propagating mode properties

In Table S1, we summarize the properties of the fundamental propagating mode in the GNR, along with properties related to the localized surface plasmon resonance of the GNR. These properties were determined by excitation with an effective δ function pulse, whose time dependence is shown in Fig. S3

Table S1: Properties of the lowest order mode of a nanowire with the same radius as the GNR.

**Parameter**	**Symbol or expression**	**Value**
Material	-	Gold
GNR length	L	150 nm
GNR radius	a	50 nm
Mode wavelength	λ	600 nm
Core dielectric const. @ 600 nm	ϵ_{co}	-8.4398
Mode effective index	$n_{\text{eff}} = \beta/k$	2.08
Plasmon lifetime	τ	4.75 fs
Plasmon decay rate	γ	210 THz
Mode round trip time	$T_L = 2n_{\text{eff}}L/c$	2.08 fs

The response of the GNR plasmon mode was calculated as depicted in Fig. S3

Dependence of results on dipole duration

The effective lifetime of the pulses used to excite the system in FDTD simulations is similar to or shorter than the plasmon lifetime of the GNR. In realistic situations, assuming the Purcell regime, the reverse is actually the case. The default optimized short pulse in FDTD

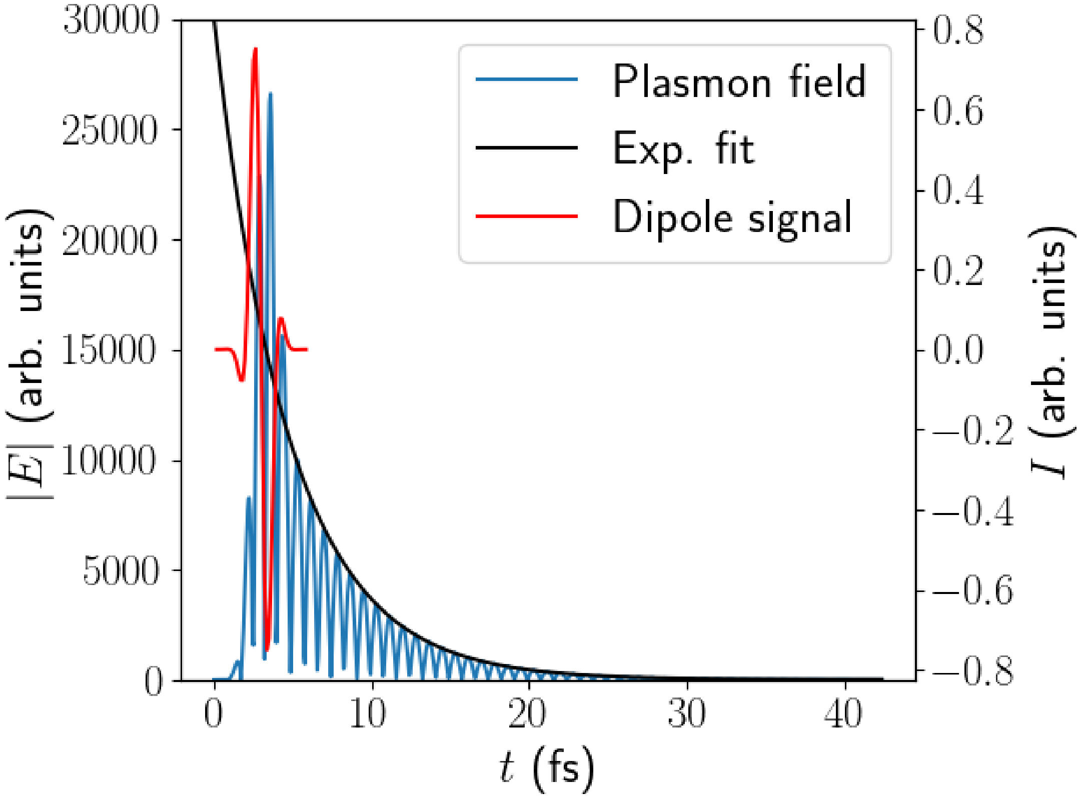

Figure S3: Time response of GNR plasmon mode after excitation by a point dipole source. The source time signal is shown in red.

simulations has a duration of approximately 4 fs. In addition, we performed simulations with longer durations of 100, 200, 300, and 400 fs, i.e. up to approximately two orders of magnitude longer than the plasmon lifetime. The results for a point dipole source positioned at the upper-right corner, are shown in Fig. S4.

As illustrated in Fig. S4a, the P_{CP} over the rod volume is almost unaffected by the dipole duration. Furthermore, Figs. S4b–f show that the spatial distribution of the DCP also remains essentially unchanged for different dipole source durations.

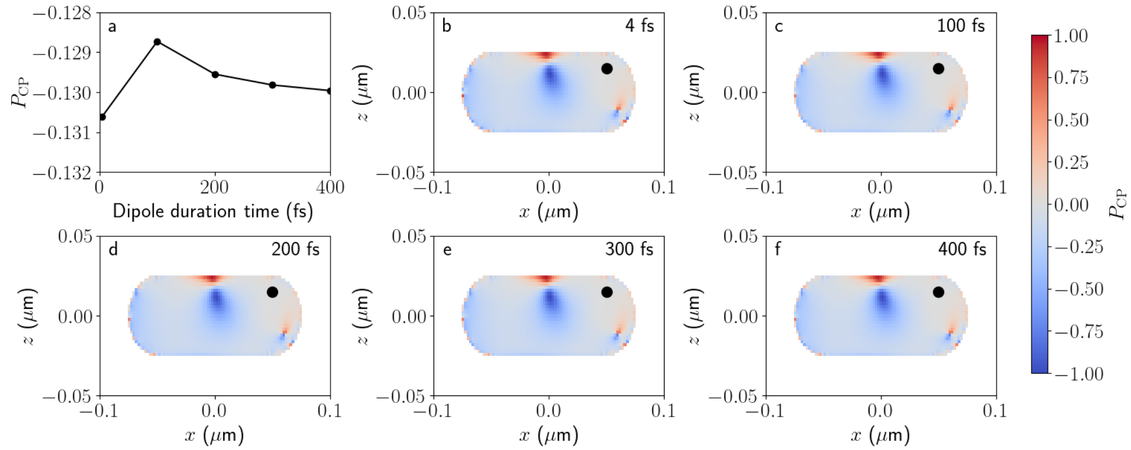

Figure S4: (a) Degree of circular polarization P_{CP} and (b–f) spatial distributions inside a 150 nm × 50 nm GNR plasmon mode excited by a point dipole source located at the upper-right corner. Results are shown for different dipole durations: (b) optimized short pulse (∼ 4 fs), (c) 100 fs, (d) 200 fs, (e) 300 fs, and (f) 400 fs.

Thus, the results for $n = 0$ are an overestimate of the DCP which in principle should tend to zero as the simulation time increases.

Effect of GNR aspect ratio on DCP of GNR dipole moment

We also investigated the P_{CP} of the GNR dipole moment at 600 nm as a function of the GNR aspect ratio (AR), as shown in Fig. S5. The case of AR = 1 corresponds to a sphere, resulting in a P_{CP} of zero. The P_{CP} reaches approximately 23% at AR = 2. Increasing the AR beyond this value does not lead to a further enhancement of DCP, ostensibly due to the fact that the mode settles into a wire mode which has no net DCP.

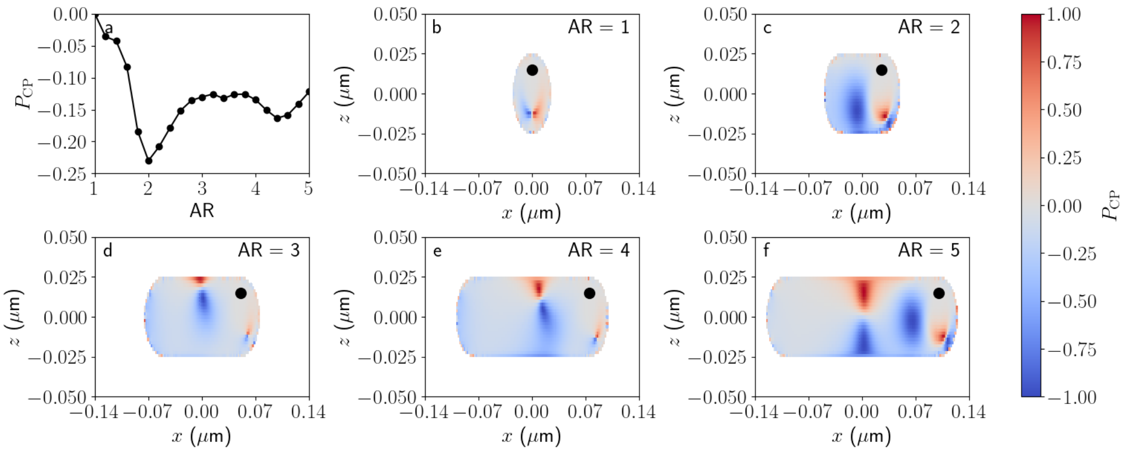

Figure S5: (a) Degree of circular polarization $P_{\rm CP}$ for different aspect ratio of GNRs ranging from 1 to 5. (b–f) spatial distributions inside the GNRs with aspect ratios 1, 2, 3, 4 and 5, respectively.

Simulation results relating to the emitted field

Scaling of emitted electric field

In Fig. S6, we present data showing that the numerically predicted fields correspond to radiation by showing that the field intensity follows an inverse square law. In this case, we have plotted the field along the y-axis. The results show an excellent fit of the function $ar^{-2} + b$ to the data, where a and b are constants, and r is the distance from the GNR center along the y-axis. This demonstrates that the calculated field in this region corresponds to emission as expected.

Maximizing the DCP of emitted light

A natural question regarding the results we report here is how large a degree of circular polarization can be achieved? A systematic exploration of this question over all experimental parameters is beyond the scope of the present paper. Instead we provide evidence below that $|P_{/rmCP}|$ can become close to unity for experimentally achievable parameters.

In particular, as shown in Fig. S7, using the same GNR dimensions as in the main paper, but lengthening the emission wavelength from 600 nm to 750 nm is enough to produce a

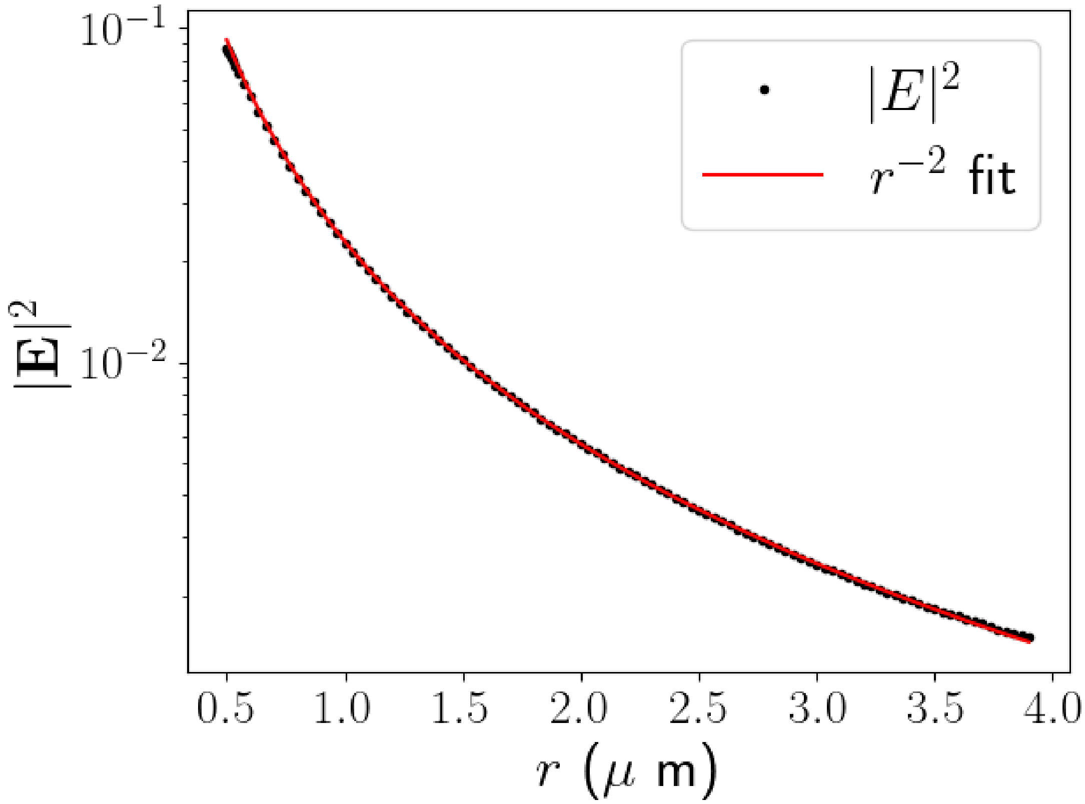

Figure S6: The simulated electric field intensity $|E|^2$ is plotted as a function of the distance from the source r along the y axis (black dots). The red line shows a fit of the function $ar^{-2} + b$ to the numerical data.

DCP for the emitted light of > 0.9 (Fig. S7(a),(b)). Interestingly, the directionality achieved for this near maximal DCP only approaches 50% as seen in Figs. S7(c),(d). The exact reason why a near perfect circular polarization does not correspond to a near perfect directionality is not yet completely clear, but is likely due to the modification of the fiber's evanescent field polarization due to the presence of the GNR.

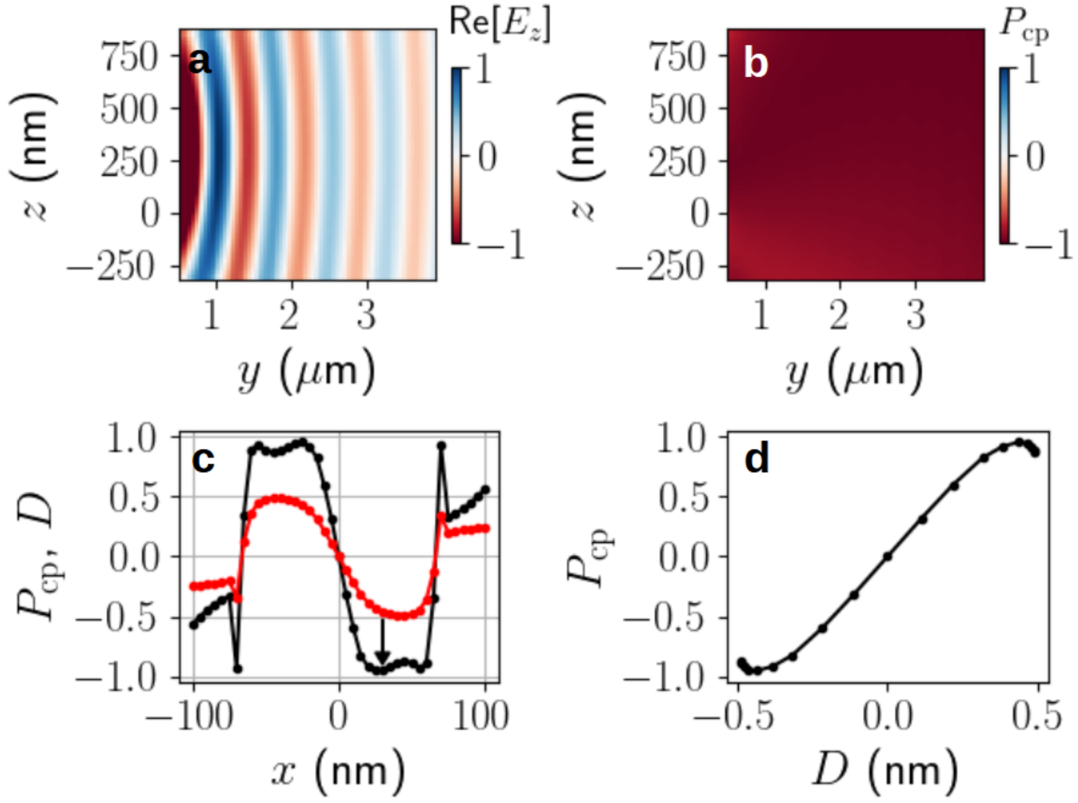

Figure S7: (a) Emission of light with almost perfect circular polarization. (b) DCP of emission shown in (a). (c) DCP of emitted light (black dots) and directionality (red dots) as a function of the dipole position. The arrow indicates the dipole position which gives the results in (a) and (b). (d) Degree of polarization P_{CP} as a function of directionality D.

Experimental methods

Here we give more details of the experimental setup, methods and analysis as introduced in the main text

Experimental setup

Our experiments take place inside a scanning electron microscope (SEM, Carl-Zeiss SUPRA 40) with a homemade fiber feedthrough.[5] An optical nanofiber fiber manufactured by a standard heat-and-pull method[6] is mounted inside the SEM and spliced to the fiber feedthrough.

By further splicing the output fiber on the air-side to a 600 nm single mode fiber (SMF) and using 600 nm long pass filters (LPF) before detection of the signal at the SPCMs, as shown in Fig. 3(d) of the main text, we ensured that the detected CL was that coupled to the fundamental mode of the fiber. For this mode, the evanescent field extends approximately one wavelength into the surrounding vacuum, and has an elliptical polarization in the $x-z$ plane dependent on the propagation direction, as shown by the thick black arrows in Fig. 3(a) of the main text. This spin-momentum locking in the evanescent field allows the conversion of polarization of CL emitted by the GNR to directionality of light in the fiber, as LCP (RCP) emission couples more strongly to the $+x$ ($-x$) propagating mode.

Details of the electron beam scan

Fig. 3(a) of the main text depicts the GNR on the fiber surface, along with the path of the electron beam, and the polarization state of the evanescent field of the fiber mode for $\pm x$ propagation. Note that the electron beam penetrates a distance δ into the fiber which is taken to be 10 nm for the GNR, and 20 nm for the silica fiber.[4]

The electron beam scan was performed at a rate of approximately 30 lines per minute, typically at a magnification between 25 and 50 times depending on the purpose of the scan. The diameter of the electron beam is approximately 5 nm, although the effective resolution of the scan is principally decided by the scan rate and SPCM output sample rate.

CL data analysis

Raw CL data was recorded as a time sequence of photon counts by SPCM1 and SPCM2. This was then reconstructed to give a CL image by aligning the data from each line scan.

The characteristic rise due to the fiber edge was used to align the data when necessary.

We note that both standard SEM images and CL images require averaging to produce low-noise images. This is performed automatically by our SEM software for standard secondary electron based images of the sample, and manually for CL images using software we developed for this purpose. Our analysis software is available at the link provided in the main text.

We calculate the directionality by calculating the ratio

$$D = \frac{I_1 - I_2}{I_1 + I_2}$$

for each point in the CL image, where I_1 and I_2 are the SPCM1 and SPCM2 photon counts respectively.

We note that which SPCM should be considered 1 and which should be considered 2 may not be clear, or can easily be mixed up by accidentally swapping the input fibers. Luckily, it is always possible to decide which is 1 and which is 2 post-measurement, since the SPCM1 end of the GNR gives larger intensities on average at SPCM1 rather than SPCM2 and vice versa. This allows the correct sign of D to be determined at analysis time for comparison with simulations.

An additional complication in calculating D is that although the relative changes in intensity at each SPCM are determined by the directionality of coupling, the *average* value of the intensity at each SPCM, which ideally should be the same, can depend on the details of individual fiber tapers, including the shape of the taper itself on each side of the nanofiber region, and any impurities introduced asymmetrically on the fiber during the GNR deposition process. It is thus necessary in general to scale the signals I_1 and I_2 so that their average values are the same. In addition, for comparison with simulations, it is necessary to set the experimental directionality for excitement at the GNR center to its ideal value of zero. For some data sets, this condition is already true to a good approximation, but for data sets where the mean values of I_1 and I_2 differ, an offset can exist at the center position. We

subtract any such offset before comparison of the 1D data (i.e. on a line through the GNR center) with simulations, so that the qualitative shape of the directionality can be compared between experiments and simulations.

References

(1) Ansys Lumerical FDTD.

(2) Haynes, W. M. *CRC handbook of chemistry and physics*; CRC press, 2016.

(3) Zarraoa, L.; González, M. U.; Paulo, Á. S. Imaging low-dimensional nanostructures by very low voltage scanning electron microscopy: ultra-shallow topography and depth-tunable material contrast. *Scientific Reports* **2019**, *9*, 16263.

(4) Uemura, Y.; Irita, M.; Homma, Y.; Sadgrove, M. Probing the local density of states near the diffraction limit using nanowaveguide-collected cathode luminescence. *Physical Review A* **2021**, *104*, L031504.

(5) Abraham, E. R.; Cornell, E. A. Teflon feedthrough for coupling optical fibers into ultra-high vacuum systems. *Applied optics* **1998**, *37*, 1762–1763.

(6) Birks, T. A.; Li, Y. W. The shape of fiber tapers. *Journal of lightwave technology* **2002**, *10*, 432–438.
